# Exploring the Optical Properties of Carotenoid-Based Nanoparticles: The Role of Terminal Groups

**DOI:** 10.3390/molecules29225456

**Published:** 2024-11-19

**Authors:** Ryuju Suzuki, Keigo Kinoshita, Takeshi Miuchi, Masayuki Nishino, Yasuhiro Shimizu, Shigeru Deguchi

**Affiliations:** 1 Research Center for Bioscience and Nanoscience, Japan Agency for Marine-Earth Science and Technology (JAMSTEC), 2-15 Natsushima-cho, Yokosuka 237-0061, Japan; 2 Department of General Engineering, National Institute of Technology, Sendai College, 48 Nodayama, Medeshima-Shiote, Natori 981-1239, Japan; 3 San-Ei Gen F.F.I. Inc., 1-1-11 Sanwa-cho, Toyonaka 561-8588, Japan

**Keywords:** carotenoids, nanoparticles, reprecipitation, color, chemical structure

## Abstract

Carotenoids are increasingly used as naturally occurring food colorants. For application as beverage colorants, fat-soluble carotenoids are formulated into dispersion systems via nanoparticle (NP) formation. In recent years, the antioxidant properties of carotenoids have gained immense recognition for their preventive health benefits, thereby highlighting further interest in their development as functional food ingredients. Although functional carotenoids in dispersion-based formulations are desirable, knowledge regarding the structural and optical properties of NPs of carotenoids other than those of β-carotene, and methods to efficiently produce and compare NPs of various carotenoids, remain scarce. In this study, NPs of β-carotene, lycopene, astaxanthin, and lutein were prepared using a simple reprecipitation method, with a focus on understanding the variations in the molecular self-assembly influenced by the quality of solvent used during reprecipitation. This study presents the novel finding that the terminal groups of carotenoids significantly affect the intermolecular interactions, thereby altering the structural and optical properties of the resulting NPs. Our findings are expected to contribute to the development of new technologies for controlling the color of carotenoids based on the crystal structure of the NPs.

## 1. Introduction

Vegetables and fruits develop vivid colors during ripening, which play a critical ecological role in their preservation. These colors attract predators and other species, facilitating seed dispersal [1,2]. Carotenoids, which are produced via the metabolism of chlorophyll during ripening, are one of the primary pigments responsible for coloration [3,4]. All carotenoid molecules possess a polyene chain comprising eight isoprenoid units, each with various terminal groups at the chain ends. The diversity of their terminal structures is vast; over 850 carotenoids have been identified in plants, animals, and microorganisms to date [5]. Because the structures of these terminal groups influence the optical properties of the carotenoid molecules [6,7], their structural diversity enables carotenoids to absorb light across the entire visible spectrum [8,9].

Hydrophobic carotenoid molecules were previously thought to be solubilized in the cell membranes [10,11]. However, research has revealed that carotenoids exist within the cytosol in the form of nanoparticles (NPs) [12,13], suggesting that the colors of ripe vegetables and fruits arise not from the carotenoid molecules themselves, but from the colors of their microcrystalline structures. Thus, the self-assembly of carotenoids and regulation of their aggregation states are vital components for the survival strategy of these plants.

In this context, Horn et al. conducted seminal studies on β-carotene NPs, which are widely used as a natural pigments in food products [14]. They prepared β-carotene NPs of various sizes via reprecipitation from isopropanol solutions of different concentrations, which exhibited hypsochromic shifts in their absorption spectra compared to the bulk crystals. Consequently, they exhibited a yellow hue, while the bulk crystals appeared red. This color shift was attributed to the differences in the molecular arrangement between the bulk crystals and NPs [15]. Subsequent studies have highlighted the pronounced influence of pH, temperature, and water content on the aggregation patterns of carotenoids in hydrated organic solvents [16,17]. Among these factors, the volume ratio of poor solvent (e.g., water) to good solvent (e.g., alcohol) is critical for controlling the molecular assembly of NPs, particularly for those prepared using the reprecipitation method introduced by Horn et al. [18]. Although precise control of the color tone is essential for the development of food colorants, knowledge of the aggregation state of carotenoids is limited, and the properties of carotenoid-based NPs are yet to be fully explored. Because minor structural variations in the carotenoid terminal groups can significantly affect the crystal structures [19,20,21,22], they can also influence the structures and optical properties of carotenoid-based NPs, thereby offering valuable insights into the ripening process of fruits and vegetables and the formation of carotenoid-based NPs.

Further exploration of carotenoids with different terminal groups has become increasingly important from a health-maintenance perspective. Carotenoids exhibit antioxidant activity by scavenging singlet oxygen and reactive radicals, thus indicating their role in preventing chronic diseases such as cardiovascular diseases, age-related macular degeneration, and cancer [23,24,25,26]. Consequently, in addition to β-carotene, compounds such as lycopene, astaxanthin, and lutein are gaining increasing attention as functional food ingredients with the potential for development into NP-based formulations. A straightforward method for producing and comparing NPs of various carotenoids is essential for the development of carotenoid-based NPs as functional food colorants. In this study, carotenoid-based NPs were prepared using the reprecipitation method, which is a simple approach for producing organic NPs, with tetrahydrofuran (THF) as the solvent. THF effectively dissolves a wide range of carotenoids at high concentrations, and is completely miscible with water. Carotenoids such as β-carotene, lycopene, astaxanthin, and lutein, were investigated for their potential use as functional food ingredients. Specifically, the effect of the water-to-THF volume ratio during reprecipitation, which is expected to influence the molecular self-assembly, on the structural and optical properties of carotenoid-based NPs was analyzed and discussed with respect to the terminal group structures.

## 2. Results and Discussion

### 2.1. Fabrication and Structural Analysis of the Carotenoid-Based NPs

**Figure 1:**
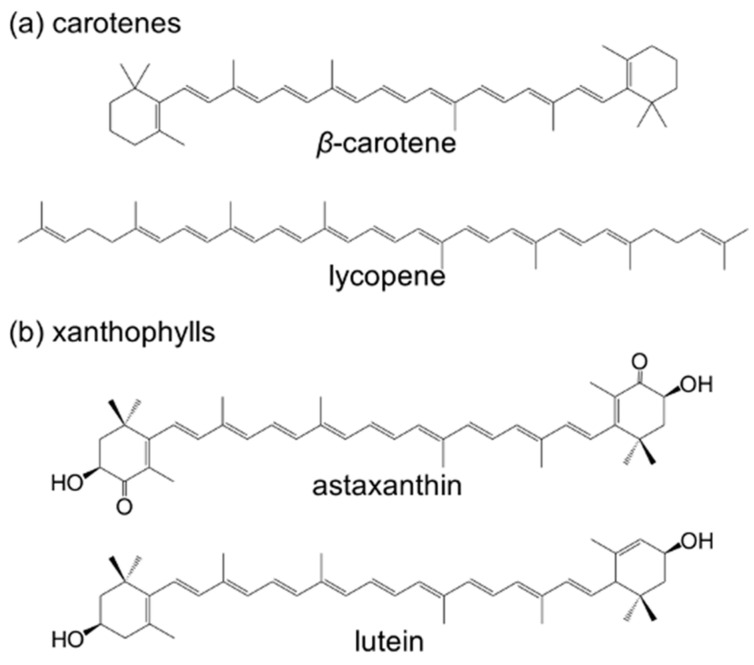
Chemical structures of the carotenoids: (**a**) β-carotene and lycopene (carotenes) and (**b**) astaxanthin and lutein (xanthophylls).

The chemical structures of the four carotenoids used in this study are shown in Figure 1. Two of them, β-carotene and lycopene, are structural isomers classified as carotenes, which solely comprise hydrocarbons (Figure 1a). Astaxanthin and lutein are classified as xanthophylls and are distinguished by their terminal groups containing hydroxyl and/or ketone functionalities (Figure 1b).

**Figure 2:**
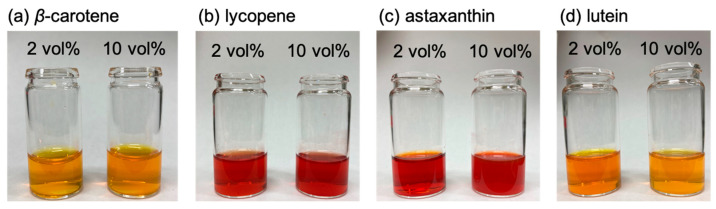
Nanoparticle (NP) dispersions of (**a**) β-carotene, (**b**) lycopene, (**c**) astaxanthin, and (**d**) lutein. The THF contents are (**left**) 2 and (**right**) 10 vol% in all the images.

**Figure 3:**
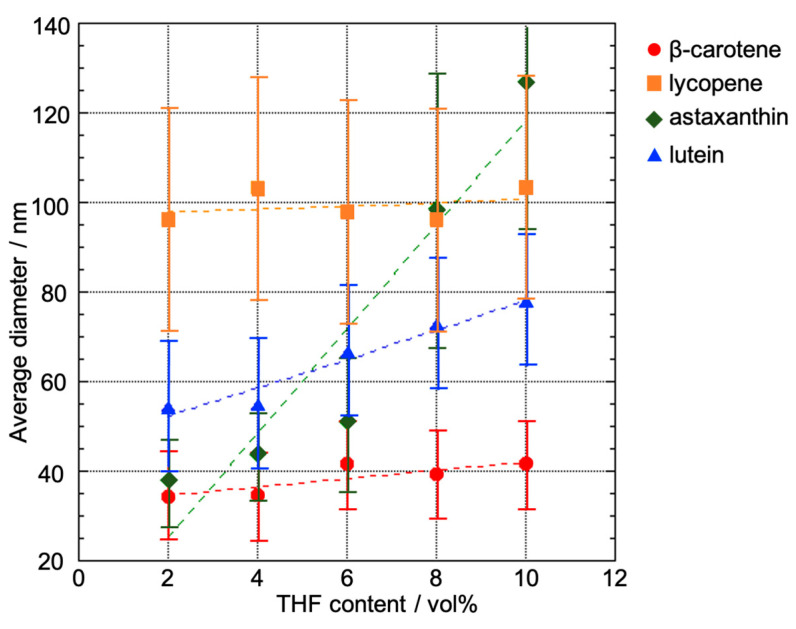
Relationship between the average size of the carotenoid-based NPs and the contents of THF used as a good solubilizing solvent in the reprecipitation method. The size of the NPs was measured by dynamic light scattering (DLS). Error bars show the size distribution.

**Figure 4:**
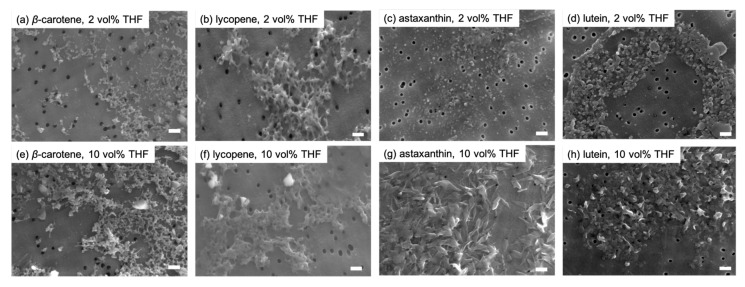
Scanning electron microscopy (SEM) images of the NPs of (**a**,**e**) β-carotene, (**b**,**f**) lycopene, (**c**,**g**) astaxanthin, and (**d**,**h**) lutein. The THF contents are (**a**–**d**) 2 and (**e**–**h**) 10 vol%. The black circles measuring 50 nm in diameter represent the pores of the filter membrane. Scale bars: 200 nm.

The reprecipitation method used to obtain carotenoid-based NPs involved dissolving the carotenoids in THF to obtain 5 mM solutions. Using this method, NPs are successfully formed with a good solvent-to-poor solvent ratio of 1 to 2 vol% [27]. An aliquot (0.2 mL) of each carotenoid in THF was added to an excess volume (9.8 mL) of water under vigorous stirring to achieve a final THF concentration of 2 vol%. The carotenoid-based NPs formed immediately and remained well dispersed in water without the use of stabilizing agents (Figure 2). The particle diameters, as determined via dynamic light scattering (DLS), was approximately 30 nm for β-carotene and astaxanthin, 50 nm for lutein, and 100 nm for lycopene (Figure 3). Scanning electron microscopy (SEM) analysis revealed that although some agglomeration occurred owing to the drying process required for sample preparation, the primary particles of each carotenoid were generally consistent with the DLS measurements (Figure 4a–d). We previously confirmed that carotenoid-based NPs prepared under similar conditions were spherical when investigated using cryo-transmission electron microscopy (cryo-TEM) [28].

The respective carotenoid-based NP dispersions in THF were prepared with final THF concentrations of 4, 6, 8, and 10 vol%. In a series of experiments, a solution of each carotenoid in THF was injected into a THF/water mixture rather than into pure water. THF in the mixture served to control the crystal growth rate during reprecipitation. The DLS results indicated that the sizes of the xanthophyll-based NPs (astaxanthin and lutein) increased with increasing THF content, whereas those of the carotene-based NPs (β-carotene and lycopene) showed no significant change (Figure 3). SEM images of the NPs obtained for a THF concentration of 10 vol% demonstrated that the carotene-based NPs maintained their shapes, as observed in the case of the 2 vol% samples, whereas the xanthophyll-based NPs exhibited well-developed facets (Figure 4e–h), which indicate that the NPs became more crystalline as THF content increased.

**Figure 5:**
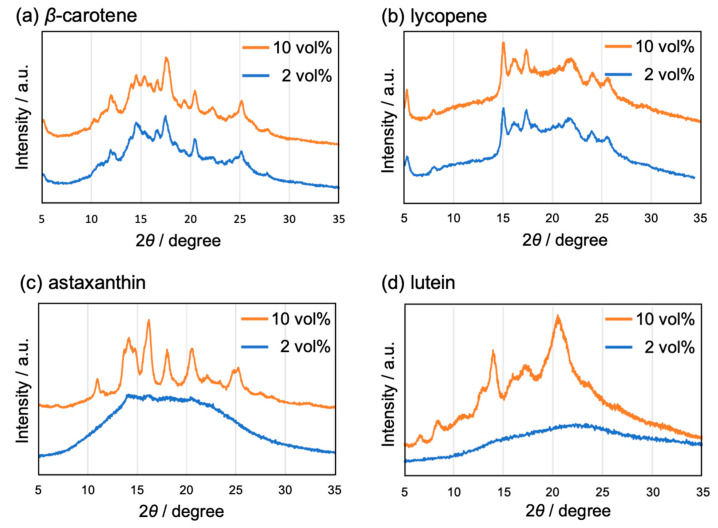
Powder X-ray diffraction (pXRD) patterns of the NPs of (**a**) β-carotene, (**b**) lycopene, (**c**) astaxanthin, and (**d**) lutein. The THF contents are 2 (blue line) and 10 vol% (orange line).

Powder X-ray diffraction (pXRD) patterns of each of the carotenoid-based NPs with THF concentrations of 2 and 10 vol% were recorded, which revealed that the XRD peaks of the xanthophyll-based NPs (astaxanthin and lutein) became clearly visible at a THF concentration of 10 vol%, indicating an increase in the crystallinity with increasing THF content (Figure 5). In contrast, the XRD peaks of the carotene-based NPs (β-carotene and lycopene) exhibited no discernible change, regardless of the THF content. The quality of the good solvent used during reprecipitation, i.e., the solubility of the target compound and its compatibility with water as a poor solvent, significantly influences the size and morphology of the resulting NPs [29]. Generally, the NP size is governed by the nucleation process, with a high degree of supersaturation favoring nucleation over crystal growth, thus resulting in the formation of smaller NPs [30]. In the present study, the solubility of xanthophylls in polar THF was higher than that of carotenes because of the presence of polar ketones and/or terminal hydroxyl groups in them. These findings suggest that an increase in the amount of THF reduces the degree of supersaturation in xanthophylls, thereby slowing the crystal growth and leading to the formation of larger and more crystalline NPs.

**Figure 6:**
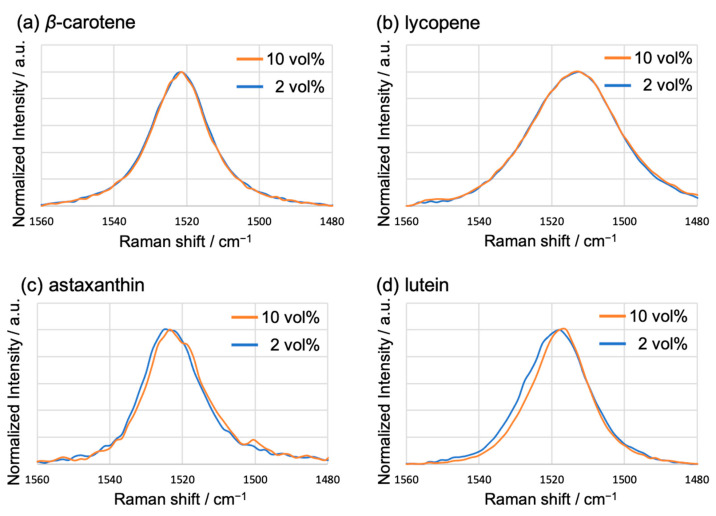
Raman spectra of the NPs of (**a**) β-carotene, (**b**) lycopene, (**c**) astaxanthin, and (**d**) lutein, displaying the ν_1_ peak corresponding to the C=C stretching vibration. The THF contents are 2 (blue line) and 10 vol% (orange line).

Carotenoid-based NPs obtained via reprecipitation exhibit a structure with a crystalline core and a surrounding amorphous shell [24]. The changes in the XRD patterns of the xanthophyll-based NPs (astaxanthin and lutein) with respect to the THF content possibly reflect a shift in the ratio of the crystalline to amorphous domains within the particles. Moreover, this variation in crystallinity influenced the conformation of the carotenoid molecules that formed these domains. Specifically, carotenoid molecules in amorphous regions are prone to molecular distortions such as rotations of the terminal rings, which were analyzed using Raman spectroscopy. A peak corresponding to the C=C stretching vibration of the polyene chain, known as ν_1_, appeared at approximately 1520 cm^−1^. It is well known that this peak shifts depending on the effective π-conjugation length of the polyene chain [31]. The shape of the ν_1_ peak of the carotene-based NPs (β-carotene and lycopene) showed no significant change regardless of the THF content (Figure 6a,b). Conversely, for the xanthophyll-based NPs (astaxanthin and lutein), the ν_1_ peak of the 10 vol% THF sample showed a reduced full width at half maximum (FWHM) and shifted towards lower wavenumbers compared to that of the 2 vol% THF sample (Figure 6c,d).

These peak shifts possibly reflect the changes in the proportions of the distorted molecules. Specifically, the smaller the strain of the carotenoid molecule, the longer the effective π-conjugation length, resulting in the shifting of the ν_1_ peak towards a lower wavenumber [31]. In our previous study, the ν_1_ peak of NPs was broader compared to that of the molecules in solution or the bulk crystals because NPs contain both distorted and undistorted molecules [28]. The proportion of molecules with varying degrees of distortions was successfully determined from the ratio of the crystalline to amorphous domains present within each carotenoid NP. The Raman spectra of the xanthophyll-based NPs (astaxanthin and lutein) indicate that the increased crystallinity with a higher THF content leads to a shift of the ν_1_ peak towards a low wavenumber because the number of distorted molecules decreases and the proportion of less distorted molecules increases. The ν_1_ peak did not exhibit any shift with increasing THF content in the Raman spectra of the carotene-based NPs (β-carotene and lycopene), which can be explained by the absence of any change in the degree of crystallinity. Notably, while pH can affect the structure of NPs formed via the reprecipitation method [16,17], its influence was not a factor in this study, as the pH of the carotenoid NP dispersions did not change with the different THF contents.

### 2.2. Optical Properties of the Carotenoid-Based NPs

**Figure 7:**
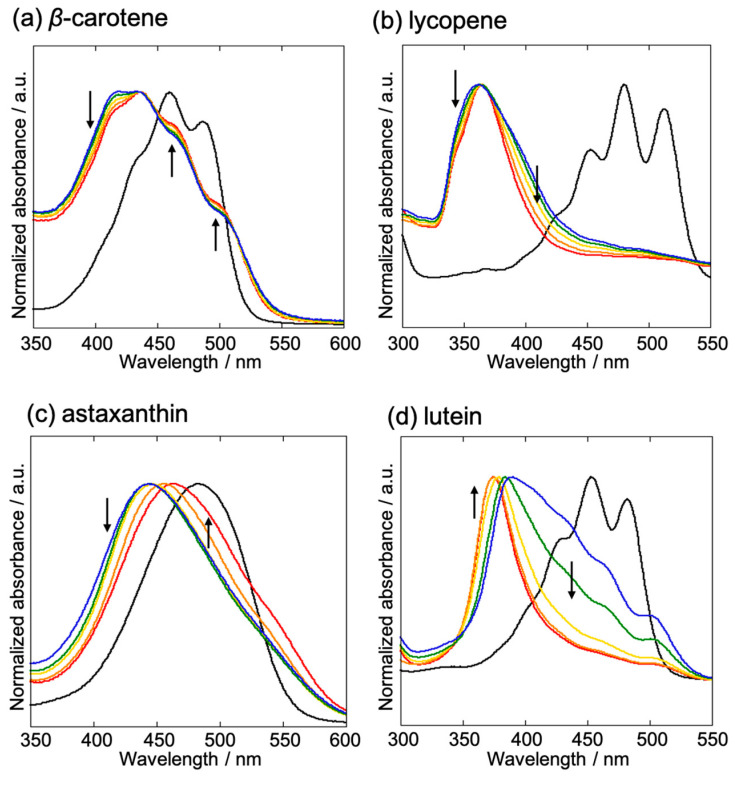
UV-vis absorbance spectra of (**a**) β-carotene, (**b**) lycopene, (**c**) astaxanthin, and (**d**) lutein. Each spectrum was recorded by varying the amount of THF used in the reprecipitation method, that is, 2 (blue), 4 (green), 6 (yellow), 8 (orange), and 10 (red) vol%. The direction of the arrow indicates the shift in the absorption spectrum with increasing THF content (2 vol% to 10 vol%). The black lines represent the spectra obtained for the respective carotenoids dissolved in pure THF.

The UV-Vis absorption spectra of the carotenoid-based NPs are shown in Figure 7. In all cases, the NPs exhibited distinctly different spectra from those of the corresponding solution states. Moreover, the absorption spectra of the carotenoid-based NPs demonstrated distinctly different patterns among themselves with varying THF concentrations.

Such absorption spectral changes in carotenoids are often classified based on the aggregate type: H- and J-aggregates [19,20,21,22]. The H-aggregates exhibit a significant blue shift and a loss of vibrational structure owing to the closely packed arrangement of parallel molecules, commonly referred to as “card-pack” aggregates, in the absorption spectrum of NPs compared to that of the corresponding monomer (i.e., the solution-state spectrum). This major spectral shift is driven by excitonic interactions between the polyene chains of the carotenoid molecules. In contrast, J-aggregates consist of loosely aligned molecules in a “head to tail” arrangement, and red shifts are observed in the absorption spectrum of NPs compared to the corresponding monomer spectrum. A similar explanation can be applied to the absorption spectra of the as-prepared carotenoid-based NPs based on the type of aggregates present.

When the THF content was varied, the absorption spectra of the carotene-based NPs (β-carotene and lycopene) exhibited significant changes, despite structural analyses using XRD (Figure 5a,b) and Raman spectroscopy (Figure 6a,b) showing no significant differences. In the case of the β-carotene NPs, it has been proposed that the NPs contain multiple small self-assemblies comprising hundreds to thousands of molecules. Horn et al. also proposed a structure in which small self-assemblies coexist with amorphous domains in gelatin-protected β-carotene NPs and discussed their absorption spectra [18]. The absorption spectra of β-carotene NPs showed a decrease in the intensity of the peak obtained at 412 nm with increasing THF content, while those of the peaks at 470 nm and 510 nm increased (Figure 7a). The peak at 412 nm was attributed to the H-aggregates, whereas those at 470 and 510 nm corresponded to the J-aggregates [17]. Therefore, the spectral changes indicated a decrease in H-aggregates and an increase in J-aggregates with increasing THF content. Within a single β-carotene NP, both H- and J-aggregates coexist within the microscopic self-assembly, and their ratio is expected to vary with the THF content.

The absorption spectrum of the lycopene NPs lacked a vibrational structure and exhibited the peak maximum at approximately 366 nm for a THF content of 2 vol%, indicating a significant shift compared to the corresponding solution-state spectrum (Figure 7b). This suggests that the NPs predominantly comprised H-aggregates. In addition, the spectral half-width decreased as the THF content increased. Molecular simulations suggest that H-aggregates tend to exhibit narrower linewidths in their absorption spectra when their assemblies are more ordered [19]. In other words, a greater molecular ordering implies a close packing of the molecules, increased self-assembly, and reduced number of aggregates. In these structures, several molecules are excited cooperatively, resulting in narrower spectral linewidths. These findings suggest that the rate of crystal growth in lycopene-based NPs during reprecipitation was moderated by an increase in the THF content, thereby resulting in the formation of more ordered H-aggregates.

β-carotene and lycopene are structural isomers with the molecular formula C_40_H_56_. However, in the case of β-carotene, the amount of H-aggregates decreased with increasing THF content, while that of the more loosely assembled J-aggregates increased. In contrast, lycopene formed more ordered H-aggregates with increasing THF content. This difference can be attributed to the presence or absence of sterically hindering terminal rings. Carotenes, solely comprising hydrocarbons, stabilize their aggregates via π–π stacking interactions between the conjugated polyene chains. The presence of terminal rings twists the planar polyene chain, thereby weakening the π–π stacking between adjacent alkene chains [20]. This terminal-ring effect was also evident in the NPs. Lycopene predominantly formed H-aggregates because the absence of terminal rings allowed highly planar molecules to interact closely. Conversely, the introduction of terminal rings into β-carotene possibly inhibited the formation of tightly bound H-aggregates, thus promoting stabilization via the formation of weakly coupled J-aggregates. Notably, although changes in the absorption spectra with varying THF content were observed, they were subtle and did not alter the visible color of the dispersion to the naked eye (Figure 2).

The position and/or shape of the absorption bands of the xanthophyll-based NPs (astaxanthin and lutein) were significantly affected by the addition of varying amounts of THF (Figure 7c,d) in contrast to those of the carotene-based NPs. The absorbance band of the astaxanthin NPs observed at 442 nm for a THF content of 6 vol% or less red-shifted to 458 nm when the THF content exceeded 8 vol% (Figure 7c). Notably, the size of the astaxanthin NPs also increased when the THF content exceeded 8% (Figure 3), presumably because the higher THF concentration affected the degree of supersaturation and the kinetics of NP formation. Furthermore, increasing the THF content affected not only the size of the astaxanthin NPs, but also their crystallinity (Figure 5c). The changes in the absorption spectra of the astaxanthin NPs can also be attributed to molecular self-assembly. Because xanthophylls contain hydroxyl groups in the terminal ring, hydrogen bonding should be considered a factor that influences aggregate formation. It has been previously suggested that hydrogen bonds play a minor role in self-assembly [21]. Presumably, the terminal ring also contains keto groups in addition to hydroxyl groups, which increase steric hindrance and inhibit π–π stacking between the polyene chains. At a lower THF content, the absorption bands appeared at shorter wavelengths, indicating the formation of H-aggregates. Under these conditions (6 vol% or less), the NPs exhibited an amorphous-like structure, suggesting that the H-aggregates in astaxanthin are metastable. Conversely, at higher THF contents, the absorption spectrum exhibited a red shift, implying the formation of J-aggregates. NPs with a high crystallinity were obtained under these conditions (>8 vol%), indicating that the J-aggregates are thermodynamically stable structures.

Interestingly, lutein NPs exhibited opposite spectral behavior to that of astaxanthin NPs with varying THF content (Figure 7d). The spectrum of lutein NPs with a final THF concentration of 2 vol% showed the peak maximum at 388 nm, with dominant vibrational structures in the longer-wavelength region. These vibrational structures disappeared and the peak maximum experienced a significant blue shift to approximately 370 nm when lutein NPs were prepared with a higher THF content (>8 vol%). Notably, visible changes in the color of the dispersion were observed with the naked eye, which became lighter as the THF content increased (Figure 2d). Lutein is known to predominantly form H-aggregates because of the strong hydrogen-bonding interactions between its hydroxyl groups [22]. Similar to the astaxanthin NPs, the lutein NPs exhibited increased crystallinity at higher THF contents (Figure 5d). However, the H-aggregates were predominant in the lutein crystals. The blue shift in the absorption spectrum and disappearance of the vibrational structure indicate that densely packed H-aggregates were formed within the crystal. As the THF content decreased, the NPs became more amorphous, suggesting that the H-aggregates were loosely packed. Previous studies have suggested that when the H-aggregates of lutein become loosely packed, vibrational structures appear with a red shift in the absorption spectrum [22]. Notably, unlike the vibrational structure observed in the absorption spectrum of β-carotene NPs, the spectrum of lutein NPs does not result from the formation of J-aggregates.

By comparing the four carotenoids, it was found that the molecular structure of the carotenoid end groups influences intermolecular interactions via two key structural factors: steric hindrance and hydrogen bonding. In turn, these affect the aggregates formed and the spectral properties of the carotenoid-based NPs. Specifically, the ratio of H-aggregates to J-aggregates appeared to vary sensitively with THF content during reprecipitation. Hydrogen bonding between the hydroxyl groups plays a crucial role in promoting the formation of H-aggregates, thereby ensuring close packing between the carotenoid molecules, as observed in the case of lutein. When lutein molecules become loosely packed due to environmental changes, they attempt to maintain H-aggregate structures despite peak shifts observed in the absorption spectrum. Conversely, some carotenoids transition to J-aggregates in response to other structural factors within their terminal rings. Steric hindrance in the terminal ring is a significant structural factor in J-aggregate formation, as observed in the case of astaxanthin, which tends to shift towards J-aggregate formation as the H-aggregates disintegrate. This steric effect is also observed in the case of carotenes that lack hydroxyl groups. For instance, lycopene, which lacks terminal rings, exhibits predominant π–π stacking between polyene chains, facilitating H-aggregate formation. However, β-carotene, with its bulky terminal rings, experiences steric hindrance that prevents H-aggregate formation, causing H-aggregates to collapse and J-aggregates to dominate. Although the prediction of NP aggregation structures solely from chemical structures is challenging because of the interplay between hydrogen bonding and steric hindrance, the reprecipitation method used in this study is a simple and versatile approach for experimentally evaluating the NP structures and optical properties of a wide range of carotenoids, many of which have significant potential for future functional applications.

## 3. Materials and Methods

### 3.1. Materials

All carotenoids (β-carotene, lycopene, astaxanthin, and lutein) were provided by San-Ei Gen F.F.I, Inc. (Osaka, Japan). THF was purchased from FUJIFILM Wako Pure Chemical Corporation (Osaka, Japan). All other reagents were commercially available and used without further purification. Water was purified to a resistance of 18.2 MΩ·cm using a PURELAB flex3 system (ELGA LabWater, Buckinghamshire, UK).

### 3.2. Fabrication of the Carotenoid-Based NPs

NPs in this study were prepared using the reprecipitation method [23]. Briefly, the carotenoids were separately dissolved in THF. Each solution (5 mM, 0.2 mL) was rapidly injected into vigorously stirred water (9.8 mL), which resulted in the formation of four distinct types of carotenoid-based NPs. The final THF concentration was 2 vol%. For the NP dispersions with varying THF contents (4, 6, 8, and 10 vol%), a THF/water mixture containing the desired amount of THF was used instead of pure water.

### 3.3. Characterization of the Carotenoid-Based NPs

The NP powders were deposited onto polycarbonate membranes via filtration (Whatman^®︎^ Nucleopore Track-Etched Membranes, Sigma-Aldrich, St. Louis, MO, USA), and the sizes and morphologies of the resulting NPs were assessed using SEM (JSM-6700F, JEOL, Tokyo, Japan). DLS measurements were performed using a dynamic light scattering spectrophotometer (FPAR-1000, Otsuska Electronics Co., Ltd., Osaka, Japan). The pXRD patterns were obtained using an X-ray diffractometer (D8 ADVANCE, Bruker, Karlsruhe, Germany). Resonance Raman spectra of the NP powders were recorded using a Raman spectrophotometer (DXR, Thermo Fisher Scientific, Waltham, MA, USA, laser wavelength = 532 nm). The UV/Vis absorption spectra were recorded using a UV-Vis spectrometer (V-570, JASCO, Tokyo, Japan).

## 4. Conclusions

The influence of the terminal structure of carotenoid molecules on the structural and optical properties of their corresponding NPs was thoroughly investigated. The reprecipitation method employed in this study enabled the fabrication of uniform NPs of four distinct carotenoids (β-carotene, lycopene, lutein, and astaxanthin). This comparative analysis revealed that the properties of carotenoid-based NPs varied depending on the terminal groups of the base carotenoid molecules. This was demonstrated by adjusting the amount of THF, which was used as the good solubilizing solvent during the NP fabrication process. In particular, the crystallinity of xanthophylls (astaxanthin and lutein), containing ketones and hydroxyl groups, increased with increasing THF content, leading to changes in particle size and shape. This variation in crystallinity altered the ratio of H-aggregates to J-aggregates in the xanthophyll-based NPs, which in turn affected their absorption spectrum. In contrast, for carotenes (β-carotene and lycopene) composed solely of hydrocarbons, changes in the absorption spectrum with THF content were observed despite no discernible structural changes in the XRD patterns or Raman spectra. This behavior, similar to that of xanthophylls, was attributed to changes in the self-assembly of carotene molecules. The self-assembly of carotenoids is influenced by two key factors: π–π interactions between polyene chains and hydrogen bonding in hydroxyl groups. This study concludes that the terminal groups of carotenoids are closely associated with each factor, thereby governing the structural and optical properties of carotenoid-based NPs. This significant discovery indicates the potential for controlling the color tone of various carotenoids and serves as the foundation for developing methods to regulate the colors of carotenoid-based NP dispersions, which are expected to be employed in future functional food applications. Moreover, this study accelerates the evaluation of the physical properties of NPs derived from a wide range of carotenoids. Our group is currently elucidating the detailed structures and coloration mechanisms of carotenoid-based NPs, and we will report our findings in due course.

## Data Availability

The original contributions presented in the study are included in the article and further inquiries can be directed to the corresponding author.
